# Eye Bleeding Due to Leech Infestation in an Ischemic Heart- Disease Patient

**Published:** 2012-04-01

**Authors:** E Shirzadeh, R Golmohammadi

**Affiliations:** 1 Department of Ophthalmology, Sabzevar University of Medical Sciences, Sabzevar, Iran; 2 Department of Anatomy, Sabzevar University of Medical Sciences, Sabzevar, Iran

**Keywords:** Leech, Eye Bleeding, Ischemic heart disease, Iran

## Abstract

A 49-year-old Iranian male with diagnosis of ischemic heart disease (IHD) and arterial hypertension (AH) was admitted at Emergency Ward of Vase’ee Hospital of Sabzevar, Iran. As well, the patient had red eye and left eye bleeding. On eye examination, leech infestation was found to be the cause of the eye bleeding. In the endemic regions in a patient presenting red eye and eye bleeding, leech infestation came up to be an important differential diagnosis. Therefore, to prevent leech infestation in endemic regions, local people are advised to be informed more effectively about the necessity to use safe, clean, and filtered drinking water, and a perfect scrutiny for leeches or other sources of infestation that should be undertaken before bathing.

## Introduction

Encyclopaedia Britannica characterizes leeches as a class of segmented invertebrates, related to earthworms. Unlike other worms, leeches have two suckers at one end and there is one small anterior sucker for feeding; also there is another larger posterior one for hanging on while they feed.[1] Leeches are primarily freshwater annelids, but some are observed to be living in the ocean and some in moist soil or vegetations.[1][2] Aquatic leeches have a worldwide distribution and it is reported that they can attach to mucosal membranes accidentally from springs and freshwater in endemic areas. They have also been described in sites like nose, tonsils, conjunctiva, pharynx/larynx, trachea/bronchi, esophagus, rectum and vagina.[3][4] Here we report an unusual case of eye bleeding due to leech infestation in an admitted ischemic heart disease (IHD) patient at an emergency ward.

## Case Report

**Fig. 1:**
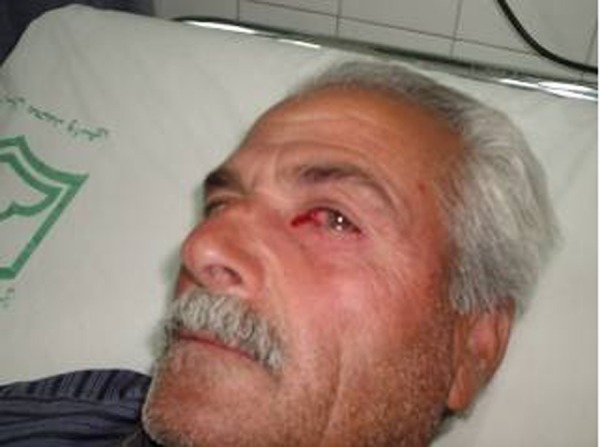
Left eye bleeding in an IHD patient.

**Fig. 2:**
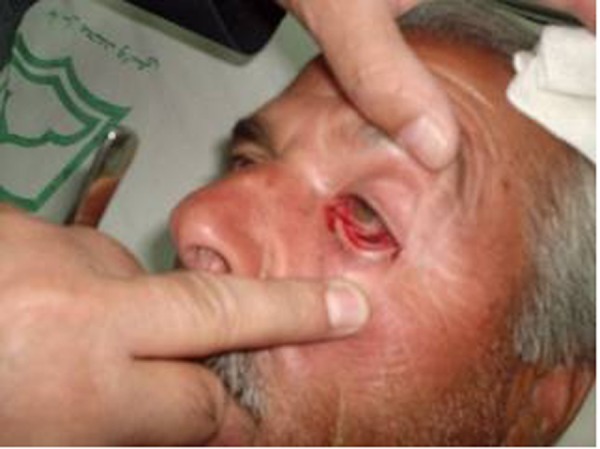
Leech in the left lower fornix.

A 49-year-old Iranian male with a history of IHD and arterial hypertension (AH) was admitted at the Emergency Ward of Vase’ee Hospital, Sabzevar, Iran. The chief complaint of the patient was red eye and left eye bleeding since a day earlier (Figure 1). The patient lived in a village. A neighbouring general physician at the nearest health centre (the first line of examination) referred the patient to the Vase’ee Hospital for controlling blood hypertension and treating the eye bleeding. The general physician’s diagnosis was eye bleeding due to AH. At hospital (the second line of examination), the on-duty general physician at the screening clinic admitted the patient with the detected diagnosis. Then, the on-call cardiologist (the third line of examination) requested a non urgent consult for evaluating the source of eye bleeding. On eye examination, visual acuity of both eyes was 20/20, both eyes had pterygium; and a dark mobile foreign body compatible with a blood-engorged live leech in the lower fornix of the left eye was detected, associated with visible bleeding (Figure 2). The leech was removed after instillation of 0.5% tetracaine eye drops via forceps. After the removal, bleeding stopped immediately. Upon history taking, the patient disclosed that he had washed hands and faces the other day with water from an open water container without watching for its content. Later on, a parasitological examination identified the foreign body most probably as a Limnatis nilotica species.

## Discussion

**Fig. 3:**
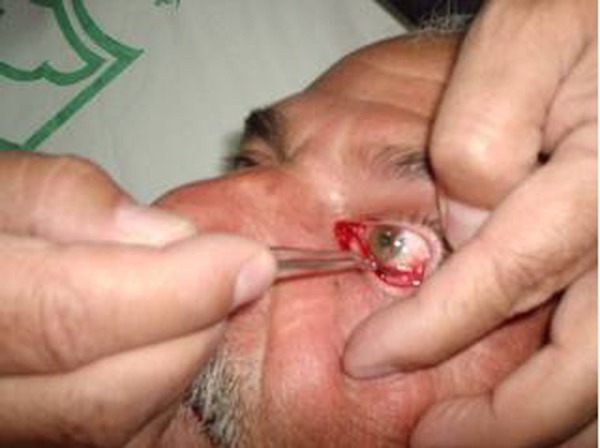
Leech removal with eye forceps.

**Fig. 4:**
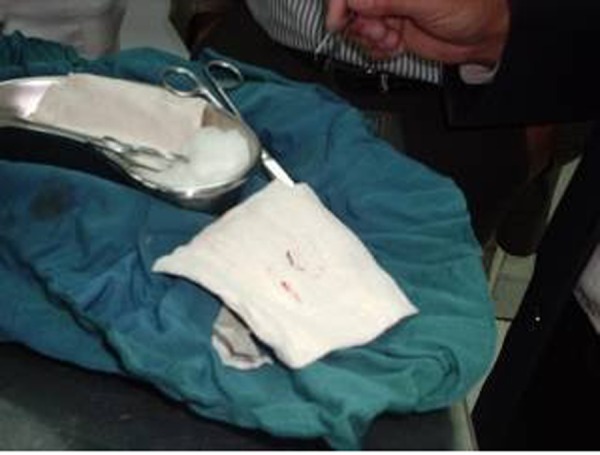
Leech after removal.

Many reports have been published on leech infestation. Leeches are reported to affect any organ system that has a natural anatomical portal of entry. Ocular leech infections have also been reported to be associated with swimming in streams and washing face with aqueduct water.[2][5][6] The presenting symptoms are varied depending on the organ system involved; epistaxis for intranasal or nasopharyngeal infestation; stridor and cough in the larynx; vaginal bleeding in genital infestation, hematuria in bladder infestation,5 and eye bleeding as in the case reported here. The appropriate treatment often involves the mechanical removal of the leech under direct vision. However, the direct removal of a leech may be difficult because of its strong attachment to the mucosal tissue as well as its sling and mobile body.[7] In the case we are reporting here, after applying 0.5% eye drops tetracaine,[2] the leech was paralyzed, and then it was easily removed with eye forceps (Figure 3 and Figure 4).

Leeches, which belong to the class Hirudinea secrete enzymes such as hirudin in their saliva that act as anticoagulants which inhibits factor IXa and thrombin, and hementerin, that is a plasminogen activator.[8] Consequently, they induce bleeding and digest considerable amounts of blood.[3] In our patient, the leech had been attached to the lower fornix of left eye for one day before coming to the health centre in the village. Leeches, once attached to the mucosa, can cause blood loss in a profound and even fatal manner,[2] especially in IHD patients. In addition to blood loss, the risks of leech infestation include allergic reactions and infection.[9] For this reason, after leech extraction, we applied eye drops of 0.5% chloramphenicol and dexamethasone four times a day. In the rural areas in Iran, bathing, drinking, or washing hands and face with spring and aqueducts water are still common, and people mostly use cupped palm of their hands to drink water or to wash their face directly from aqueduct water or springs. This is suspected to cause leech infestation in humans. However, unlike oronasal infestation, ocular leech infestation is reportedly rare.

Regarding the differential diagnosis, upon history taking, the most probable diagnosis in our patient was subconjunctival hemorrhage due to AH and IHD. However, eye examination revealed eye bleeding instead of subconjunctival hemorrhage. Therefore, in the endemic regions in a patient presenting red eye and eye bleeding, leech infestation came up to be an important differential diagnosis. Eye bleeding due to ocular trauma and scleral rupture due to glaucoma for iris prolepsis was ruled out of the history, eye examination, and normal intraocular pressure. Red eye due to common causes including pterygium and systemic diseases such as diabetic mellitus could be excluded by clinical findings and laboratory examinations too. This patient had pterygium, and his laboratory examinations, including fasting blood sugar were normal but he was an IHD case and a candidate of coronary artery bypass.

In conclusion, ocular leech infestation is considered as one cause of eye bleeding in the reported case; therefore, to prevent leech infestation in endemic regions, local people are advised to be informed more effectively about the necessity to use safe, clean, and filtered drinking water, and a perfect scrutiny for leeches or other sources of infestation should be undertaken before bathing. Also, both local and government officials need to support health education and to facilitate public access to safe water.
